# Mr. Hill, on the Liverpool Controversy

**Published:** 1809-05

**Authors:** G. N. Hill

**Affiliations:** Chester


### *To the Editors of the Medical and Physical Journal.*

"All that is wanting to the perfection of this art will undoubtedly be found, if able men, and such as are instructed in the ancient rules, will make a farther enquiry into it, and endeavour to arrive at that which is hitherto unknown, by that which is already known."

HIPPOCRATES, Oper. Passim.

GENTLEMEN,

THE life of a medical man may not inaptly be compared to that of a navigator, doomed, from certain inevitable circumstances, to traverse seas concealing dangerous rocks, treacherous sand banks, or insidious currents, amongst which, when on duty, he is almost momentarily compelled to avail himself of any friendly beacon which presents itself in aid of the invaluable chart to conduct him in his perilous course through the mighty waters. Still, notwithstanding every suggestion of human prudence, disasters will occur; but whenever they unavoidably happen, instead of discouraging, only animate the brave and persevering to new exertions, unwearied diligence, and greater circumspection upon future trying occasions. That the parallel holds good the faculty at large have had sufficient proof, upon a late melancholy occasion which took place in Liverpool. You have doubtless read the Trial of a Charles Angus, of that town, for murder, at Lancaster, in September last, and have probably since seen an able "Vindication" of the evidence given upon this awful investigation, by the highly respectable medical witnesses in behalf of the prosecution.

Notwithstanding the seeming contradiction involved in the two following quotations, from the "Vindication," I have ventured to hazard a few remarks, through the medium of your valuable publication, upon some of the circumstances cumstances attending the dissection of the miserable object supposed to have been murdered, conformably to the following general invitation, (given at p. 86—87. "And with reference to the future, we feel ourselves justified in calling upon our brethren to give this important subject all the attention and consideration it requires;) and under a full conviction that an inspection of greater importance to the cause of truth and medical testimony scarce ever happened; undismayed by the subsequent declaration, (at p. 87, "we shall take no notice of any reply which may be made to this pamphlet, publicly or otherwise"); it will at once be obvious that the thoughts which occurred to me upon its perusal, though not unimportant, require no replication, being such as will, no doubt, occur to many others. The general tenor of the "Vindication," proves it to have been drawn up in the spirit of truth and justice, it is therefore impossible to conceive, that the highly respectable authors can be offended at any fair remarks arising from an attentive perusal of this very proper and judicious document, more especially if such remarks can either tend to the further illustration of the case in question, or lead to a more extended examination of any future one similarly situated.

We are enjoined by some of our highest and most respectable medical authorities, never to omit the scrupulous reviewal of every ambiguous case brought to a mortal close, in order, if practicable, to discover if we have been misled by appearances; if an omission of duty, or commission of error, can unfortunately through any inadvertence, attach themselves, it being an evidence of wisdom to retrace our steps upon every momentous occasion, that we may be the better enabled to govern our future conduct. From the circumstances detailed at the trial, it is probable that the unhappy woman swallowed the fatal poison on the Wednesday morning fasting, which circumstance, added to that of her being pregnant, would occasion an instantaneous and violent rejection of the offending matter. Now, on the supposition that this offending matter was a solution of muriate of mercury, or any equally virulent poison, it is not possible to conceive how it could pass the œsophagus without commencing its depredations upon that animated tube, it being well known that all substances permitted to pass from the mouth to the stomach, are not conveyed thither by the single gulp, sensibly experienced as the first act of swallowing, but by a series of similar successive actions they ultimately arrive at the destined cavity, that is, by alternate alternate contraction and expansion, acting upon the descending substance; it is likewise a fact, that this organized passage is the seat of the principal glands furnishing the succus gastricus, and not so destitute of sensibility to the influence of an active agent, as might to ignorant or inattentive persons upon superficial reflection appear; inattention it can alone be, when it is considered how few escape with impunity, when attempting to swallow substances of an angular form, too large, too hot, acrid, spirituous, &c. Hence, although less highly organized than the stomach, of which it is a continuation, yet it is sufficiently so to be sensible of considerable impression from the contact of an active poison, which had not only passed, but repassed it by violent regurgitation. Now this being the point of fact, it becomes a very natural question, why was not the œsophagus comprehended in the dissection? why was it not opened from the stomach to the mouth? A matter so inimical to the stomach as some agent undeniably appears to have been, could not have travelled the road to it and back again independent of a traceable impression marking its rout; let any sceptic take a mouthful of a weak solution of muriated mercury, and he will not be long in acknowledging its power, without extending the experiment to an act of deglutition.

To some superficial reasoners, these remarks may appear trivial, but no circumstance that can contribute in the slightest degree to the elucidation of so serious a case, and which may not be the last, will fairly and honestly be so designated, especially when the inestimable value of collateral proof is justly appreciated; but indeed any frivolous remark of this nature is at once repelled, by observing, that had the œsophagus been examined, and upon due inspection, presented any appearances differing from those found subsequent to excessive vomitings, where no poison had been swallowed, such appearances would have erected an irrefutable argument against destruction of the stomach by the gastric juice. After corroborative proof like this, it can scarcely be credited, that any man claiming the slightest pretensions to knowledge in medical science, would have had the hardihood to advance so absurd an opinion; it is undeniable that the instant a mortal poison became closely applied to the parts through which it had to pass from the back of the tongue to the fundus of the stomach, all such parts would be more or less affected by the deadly approximation, added to the efforts of nature to repel the horrid attack; which salutary efforts, consisting of retrograde action, must add to the proximity of contact between the offending matter and the parts offended, until ejection was completed. In the case of Miss Burns, the perverted motion was nearly incessant, and was no doubt continued long after the entire expulsion of the obnoxious substance, according to a well known law of the animal œconomy; I presume then to say, that no part, directly or indirectly implicated in the extensive derangement found in her stomach, should have been left unexplored; as to expecting to find any thing in this cavity, which by a chemical test would lead to detection of the exact nature of the substance imbibed, the idea was fallacious, (although to subject the contained fluid to every feasible experiment was an indispensible duty) because from the immediate rejection, incessant vomiting, and continued dilution, there is scarce a probability that a particle ever reached, much less passed the pylorus; the sum of the effects of poisonous contact, inordinate action for near 50 hours, and universal exhaustion, form an aggregate of evil fully adequate to the ruinous appearances discovered without any man attempting to stagger credibility by even a bare allusion to destruction by the gastric juice; whenever this rare phenomenon has taken place, the viscus was never found to have been previously excited into a state of long continued enormous preternatural action, highly prejudicial, if not wholly destructive of its performance of the office of an healthy secreting organ. Farther, it may be just observed, *(en passant)* Do all secreted animal fluids become more active in proportion as they are redundantly and prematurely secreted? After being inordinately emulged for two days and nights, would what gastric juice was left in the stomach, if any was, be in a state of sufficient activity, upon nature giving up the struggle, to destroy its coats to the extent found, or to any extent at all? The last effort to vomit was equal to the production of the hole, and which, as the operations of nature never stand still, would not be contracted but enlarged by death. Lastly, the great debility evinced by the remission of the violent efforts by the dyspnœa, by the comparative repose, and by the commencement of diarrhœa, are all damning proofs against the capability of the stomach to furnish the means of its own organic destruction.

The operation of opening a dead body for the purposes of a legal decision, upon the causes of death, is precisely that situation of all others which requires the most patient scrutiny of the medical artist, and when called upon to give evidence, not the smallest parade of learning, or ostentation tentation of science, should appear. "Be the plainest men in the world in a court of justice," says the venerable Blizard, "never harbour a thought, that if you do not appear positive, you must appear little and mean ever after; many old practitioners have erred in this respect. Give your evidence in as concise, plain, and yet clear a manner as possible; be intelligent, candid, open, and just, never aiming at appearing unnecessarily scientific; state *all the sources* by which you have gained your information; if you can, make your evidence a self-evident truth; thus, though the court may at the time have too good or too mean an opinion of your judgment, yet they must deem you an honest man; never then be dogmatic, or set yourselves up for judge and jury; take no side whatever; be impartial, and you will be honest. In courts of judicature, you will frequently hear the counsellors complain when a surgeon gives his opinion with any the least kind of doubt, that he does not speak clearly; but if he is loud and positive, if he is technical and dogmatic, then he is allowed to be clear and right. I am sorry to have to observe, this is too frequently the case."

Now, upon reviewing the case of Miss Burns according to the maxims just quoted, wherein have the able practitioners employed, appeared to deviate from the instructions of the anatomical teacher, in not gaining possession of *all the sources* of information which might have been obtained, the more perfectly to enable them to prevent the origin of any sophistical, idle, or unfounded conjectures, which might be attempted to be established from motives of a nature too unworthy to be mentioned? "We did not examine the ovaria at the time that the uterus was taken out of the body, *because we were perfectly satisfied in our minds with the other proofs* which we found of pregnancy." Vindic. p. 22, note. The hurry and anxiety too generally attending the opening of bodies for an inquest, added to their being often examined under the influence of preconceived opinion, afford circumstances highly prejudicial to correct investigation, and the most perfect illustration a case will often admit, when examined more coolly, and under a different situation. The observations respecting the œsophagus, which have been (perhaps presumptively) offered in this paper, apply with double force to the appendages of the uterus, an organ of such relative importance in the female system, as upon every occasion entitles it to the utmost attention; but whilst it is conceded, that the appearances presenting themselves immediately upon opening this viscus, were so striking as to carry instant conviction to the minds of the intelligent spectators, of the truth of what had recently happened; yet the acknowledged omission respecting the non-examination of the ovaria cannot be justified.

If, as is observed by Dr. Denman, and which is the anatomical fact, that the corpora lutea "are largest and most conspicuous in the early state of pregnancy, and remain for sometime after delivery, when they *gradually* fade and wither till they disappear;) was it not of the very highest moment, that this body should have been one of the first explored? Immediate inspection would have demonstrated how far it had advanced to a state of withering and decay; hence it is clear, that had it been a corpus luteum, the result of an old impregnation, it would have been found in a correspondent faded state; but here, even at the distance of six months, one of these bodies is discovered, and which, to the discoverers, presents itself in proof of a late impregnation. "Upon examining the uterus *since* the trial, a corpus luteum has been found in one of the ovaria, which is a proof beyond all contradiction, that she had once at least been pregnant; nothing can account for the corpus luteum in the ovaria but impregnation," Vind. p, 30—31. Thus is learnt the high value of knowledge acquired at the natural and proper point of time; it is devoutly to be hoped, that this memorable instance will for ever operate as a friendly beacon to warn the advancing generation of anatomists and surgeons, against a hasty or superficial dissection of a dead body; many causes have concurred to produce a slight examination of this kind, very innocently, and sometimes unavoidably, but perhaps never independently of serious injury to the discovery of truth.* When it is considered how rarely medical men are enabled to speak decisively in courts of justice, as they are ignorantly and unreasonably expected to do, we certainly cannot be too circumspect, in obtaining

* Surgeons in general are much too prone to precipitation upon these occasions, to carry the knife hastily to the spot where the cause of evil seemed to exist, and to that exclusively, frequently overlooking those situations where the cause of death might have been found, thus disqualifying themselves for a ready reply when asked in a court of justice. "Did you examine the head, thorax, belly, &c. &c.?" Farther comment upon superficial examinations is necessary, when it is remembered that the immediate cause of obstruction is not always to be found where distressing symptoms during life appeared most urgent.

ing every particle of information *before* the day of trial, which the subject of investigation will yield; coming thus prepared, no hesitating, clashing, contradictory evidence could possibly maintain any stand in the minds of twelve honest men; for in the emphatic words of the great anatomist before quoted, "surgeons come into a court of justice for what? not to declare this or that thing, on any side of any question, or for or against any party of men; No! if you are not the advocate for mere simple truth, go not near the place." In the case of Miss Burns, considerable stress was laid upon the supposed offender having been possessed of a curious instrument, which it was conjectured was calculated in certain hands to effect a mechanical action upon the os uteri; it might therefore be reasonably enquired of the dissectors, whether this part of the uterus presented any other appearances of local injury than what might be supposed to have arisen from the expulsion of the fœtus? was the examination extended to the ascertainment of this point. Much was said upon the size of the uterus when first exposed to view. A few days since I saw a female whom I have delivered of several children; she frequently had considerable sanguineous evacuations; during, and immediately after her labours, it is my practice instantly upon the expulsion of a child from the womb, to direct the abdomen of the mother to be gently and equably pressed over this viscus, by the warm hands of an assistant; upon two occasions this was not done so quickly or perfectly as I wished in the first instance, but upon being performed to my satisfaction, a current of air rushed per vaginum, which astonished me for the moment. Now whence this air came from, or how it so expeditiously occupied the uterus, is a curious matter; but in a case where death was about to take place from other causes than hæmorrhage, it is easy to conceive, that upon no abdominal pressure being used, especially after the premature evacuation of a fœtus, such a volume of air might enter the uterus as to keep it distended in the manner found; the relaxed state of the viscus, from obvious causes rendering it incapable of acting upon its new occupant.'

I am, &c.

G. N. HILL.

*Chester, November* 30, 1808.

